# Facet-dependent electrooxidation of propylene into propylene oxide over Ag_3_PO_4_ crystals

**DOI:** 10.1038/s41467-022-28516-0

**Published:** 2022-02-17

**Authors:** Jingwen Ke, Jiankang Zhao, Mingfang Chi, Menglin Wang, Xiangdong Kong, Qixuan Chang, Weiran Zhou, Chengxuan Long, Jie Zeng, Zhigang Geng

**Affiliations:** Hefei National Laboratory for Physical Sciences at the Microscale, Key Laboratory of Strongly-Coupled Quantum Matter Physics of Chinese Academy of Sciences, Key Laboratory of Surface and Interface Chemistry and Energy Catalysis of Anhui Higher Education Institutes, Department of Chemical Physics, University of Science and Technology of China, Hefei, Anhui 230026 P. R. China

**Keywords:** Electrocatalysis, Electrocatalysis, Catalytic mechanisms, Reaction kinetics and dynamics

## Abstract

The electrooxidation of propylene into propylene oxide under ambient conditions represents an attractive approach toward propylene oxide. However, this process suffers from a low yield rate over reported electrocatalysts. In this work, we develop an efficient electrocatalyst of Ag_3_PO_4_ for the electrooxidation of propylene into propylene oxide. The Ag_3_PO_4_ cubes with (100) facets exhibit the highest yield rate of 5.3 g_PO_ m^−2^ h^−1^ at 2.4 V versus reversible hydrogen electrode, which is 1.6 and 2.5 times higher than those over Ag_3_PO_4_ rhombic dodecahedra with (110) facets and tetrahedra with (111) facets, respectively. The theoretical calculations reveal that the largest polarization of propylene on Ag_3_PO_4_ (100) facets is beneficial to break the symmetric π bonding and facilitate the formation of C-O bond. Meanwhile, Ag_3_PO_4_(100) facets exhibit the lowest adsorption energies of ^*^C_3_H_6_ and ^*^OH, inducing the lowest energy barrier of the rate-determining step and thus accounting for the highest catalytic performance.

## Abstract (web summary)

The electrooxidation of propylene into propylene oxide is important in many industrial sectors. Here, the authors demonstrate efficient and facet-selective formation of propylene oxide using Ag_3_PO_4_ cubes and investigate the reaction mechanism.

## Introduction

Propylene oxide (PO) is an important industrial intermediate that can be transformed into various commodity chemicals such as polymers, propylene glycol, polyurethane foams, propylene carbonate, and so on^1–3^. Common industrial methods of PO production include chlorohydrin process, Halcon process, directed oxidation process, and hydrogen peroxide-based process (HPPO)^4^. The chlorohydrin process requires abundant environmentally hazardous chlorine and produces a large amount of sludge^5^. The Halcon process involves autoxidation of ethylbenzene or isobutene to produce alkylhydroperoxide that acts as an oxidant to produce PO but accompanies with the substantial formation of peroxycarboxylate^6,7^. The directed oxidation process needs to be operated at high temperature, leading to the formation of byproducts such as acrolein and CO_2_^8–10^. The HPPO has been restricted to manufacture PO from propylene due to the high cost and insufficient supply of H_2_O_2_^11–13^. Based on the aforementioned analysis, there is an urgent demand to develop an environmentally friendly, low-cost, and simple process to achieve a highly efficient synthesis of PO.

The electrooxidation of propylene into PO has attracted particular attention. This process utilizes sustainable and abundant water as an oxygen source under ambient conditions, with pure hydrogen generated on the counter electrode simultaneously^14–16^. Moreover, the mild reaction conditions require less handling and distribution infrastructure than those are necessary for PO production^17^. Currently, various catalysts have been applied to the electrooxidation of propylene^18–20^. For instance, Chorkendorff et al. prepared a Pd electrode that exhibited catalytic performance for the electrooxidation of propylene^21^. Since Pd favored the activation of C-H bond on allyl carbon, leading to the production of acrolein, the selectivity for PO was as low as 25%. Ag-based materials preferred to activate the C=C double bond rather than methyl hydrogen (α-H) in propylene, benefiting the formation of PO^8,9,22,23^. Holbrook et al. reported that PO was directly obtained via the electrooxidation of propylene on a silver electrode, but suffered from low activity (<0.01 g_PO_ m^−2^ h^−1^)^24^. For high yields of PO, it would be accessible if we modify Ag-based catalysts to enhance their activity.

A typical route to engineer the structures of Ag-based catalysts is based on regulating the exposed facets. The catalytic performance for the electrooxidation of propylene can be described by the adsorption energies of propylene (*E*_ads.Pr*_) and oxygen species (*E*_ads.O*_)^25–27^. Since *E*_ads.Pr*_ and *E*_ads.O*_ are sensitive to the facets, optimizing the catalytic performance requires the construction of uniform facets. Ideal facets should exhibit specific density of states (DOS) that properly overlap with the *p* orbitals of adsorbed propylene (Pr^*^) and oxygen species. Moreover, breaking the symmetric π bonding for the activation of C=C double bond demands the degree of propylene polarization that is also dependent on the facets. Therefore, fabricating uniform facets of Ag-based catalysts that are optimized by adjusting the DOS and propylene polarization serves as a promising way to promote the catalytic performance for the electrooxidation of propylene.

Herein, we developed highly efficient electrocatalysts of Ag_3_PO_4_ cubes that performed a high yield rate of PO production for the electrooxidation of propylene. Three types of Ag_3_PO_4_ crystals enclosed by (100), (110), and (111) facets were fabricated, including Ag_3_PO_4_ cubes, rhombic dodecahedra, and tetrahedra, respectively. During the electrooxidation of propylene, Ag_3_PO_4_ cubes exhibited the highest yield rate of 5.3 g_PO_ m^−2^ h^−1^ in 0.1 M phosphate buffer solution (PBS) at 2.4 V versus reversible hydrogen electrode (vs RHE), which was 1.6 and 2.5 times higher than those over Ag_3_PO_4_ rhombic dodecahedra and tetrahedra, respectively. Based on density functional theory (DFT) calculations, the formation of bidentate CH_3_CHCH_2_OH^*^ (PrOH^*^) intermediate from Pr^*^ and ^*^OH was the rate-determining step (RDS). The activation barrier for the formation of PrOH^*^ on (100) facets was 1.27 eV, which was lower than those on (110) and (111) facets of Ag_3_PO_4_. The lowered *E*_ads.Pr*_ and adsorption energies of OH^−^ (*E*_ads.OH*_) over Ag_3_PO_4_ cubes were favorable to the activation of propylene, resulting in the enhanced activity relative to Ag_3_PO_4_ rhombic dodecahedra and tetrahedra.

## Results

### Preparation and characterization of Ag_3_PO_4_ crystals

**Fig. 1:**
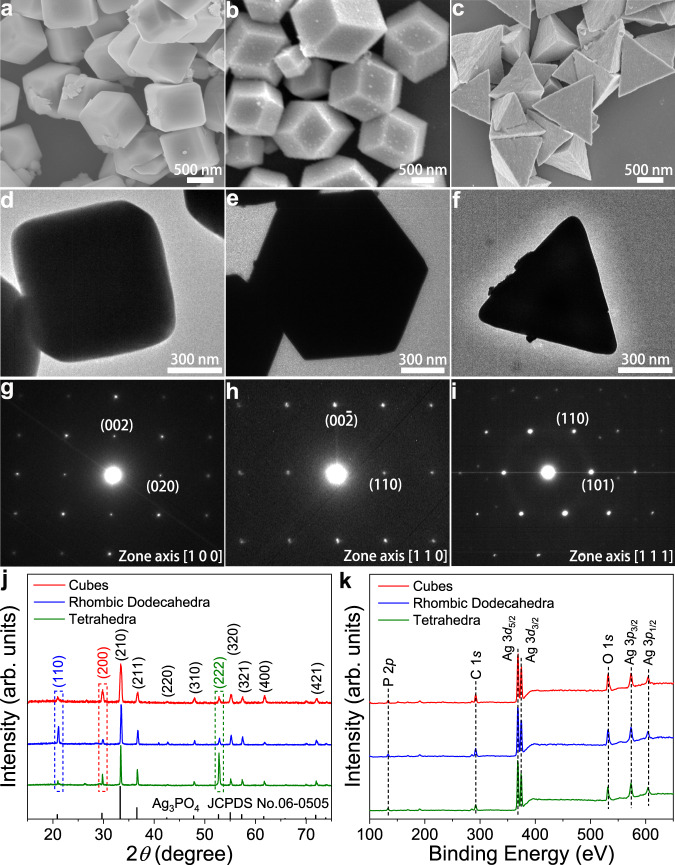
Structural characterizations of Ag_3_PO_4_ crystals. **a**–**c** SEM images of Ag_3_PO_4_ cubes (**a**), rhombic dodecahedra (**b**), and tetrahedra (**c**). **d**–**f** TEM images of a Ag_3_PO_4_ cube (**d**), rhombic dodecahedron (**e**), and tetrahedron (**f**). **g**–**i** SAED images of a Ag_3_PO_4_ cube (**g**), rhombic dodecahedron (**h**), and tetrahedron (**i**). **j**, **k** XRD patterns (**j**) and survey XPS spectra (**k**) of Ag_3_PO_4_ cubes, rhombic dodecahedra, and tetrahedra.

Typically, Ag_3_PO_4_ crystals with different exposed facets were prepared via solvent-phase synthesis under ambient pressure at room temperature^28,29^. As shown in the scanning electron microscopy (SEM) and transmission electron microscopy (TEM) images, Ag_3_PO_4_ crystals exhibited uniform morphologies of cubes, rhombic dodecahedra, and tetrahedra, respectively (Fig. 1a–c). The average edge length of Ag_3_PO_4_ crystals was around 750 nm (Fig. 1d–f). The SEM images and the corresponding energy-dispersive X-ray spectroscopy elemental mapping for Ag_3_PO_4_ crystals show the homogeneous distribution of Ag, P, and O elements throughout the whole structure (Supplementary Fig. 1). Figure 1g–i displays the selected area electron diffraction (SAED) patterns of Ag_3_PO_4_ crystals. The SAED pattern of an individual Ag_3_PO_4_ cube displayed (002) and (020) facets with a [100] zone axis. In addition, the SAED patterns of Ag_3_PO_4_ rhombic dodecahedron and Ag_3_PO_4_ tetrahedron were recorded along the [110] and [111] direction, implying that the exposed surface of Ag_3_PO_4_ rhombic dodecahedron and tetrahedron consisted of (110) and (111) facets, respectively^30^. To further confirm the dominated (100), (110), and (111) facets in the as-prepared Ag_3_PO_4_ cubes, rhombic dodecahedra, and tetrahedra, respectively, we conducted X-ray diffraction (XRD) measurements. As shown in Fig. 1j, all of the Ag_3_PO_4_ crystals displayed the characteristic peaks located at 20.9°, 29.7°, 33.3°, 36.6°, 42.5°, 47.8°, 52.7°, 55.0°, 57.3°, 61.6°, and 71.9°, which were attributed to the (110), (200), (210), (211), (220), (310), (222), (320), (321), (400), and (421) facets of the body-centered cubic structure of Ag_3_PO_4_ (JCPDS No. 06-0505)^31^. Based on the peak intensity of main diffractions of (200), (110), and (222) facets, the peak–intensity ratio of (200)/(110)/(222) for Ag_3_PO_4_ cubes, rhombic dodecahedra, and tetrahedra were calculated to be 1.85:0.99:1.00, 0.78:2.55:1.00, and 0.36:0.15:1.00, respectively. This result further indicates that the primarily exposed facets of Ag_3_PO_4_ cubes, rhombic dodecahedra, and tetrahedra are (100), (110), and (111) facets, respectively (Supplementary Table 1). To investigate the composition of Ag_3_PO_4_ crystals, we carried out X-ray photoelectron spectroscopy (XPS) measurements. Figure 1k shows the survey XPS spectra of Ag_3_PO_4_ crystals. The characteristic peaks at around 134, 285, 368, 374, 532, 574, and 604 eV were observed, which were attributed to P 2*p*, C 1*s*, Ag 3*d*_5/2_, Ag 3*d*_3/2_, O 1*s*, Ag 3*p*_3/2_, and Ag 3*p*_1/2_, respectively^32–34^. These results indicate that Ag_3_PO_4_ crystals possess the same chemical compositions.

### Catalytic performance of Ag_3_PO_4_ crystals for the electrooxidation of propylene

**Fig. 2:**
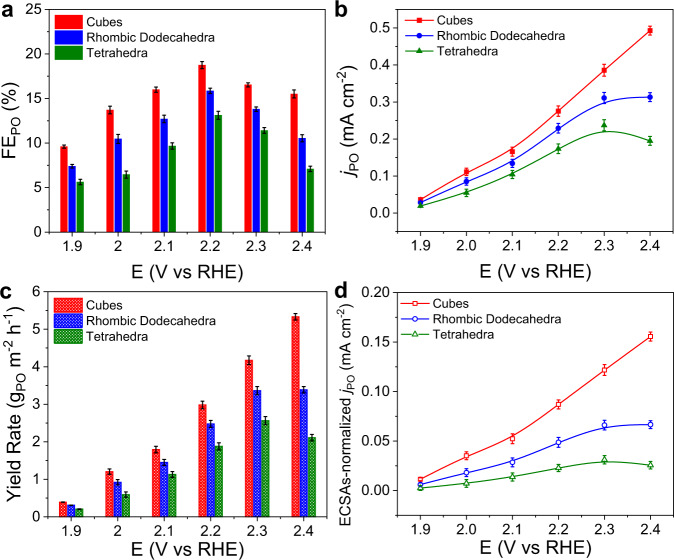
Catalytic performance of Ag_3_PO_4_ crystals for the electrooxidation of propylene. **a**–**d** FE_PO_ (**a**), *j*_PO_ (**b**), yield rates of PO (**c**), and ECSAs-normalized *j*_PO_ (**d**) over Ag_3_PO_4_ crystals. The error bars represent the standard deviations for the three independent measurements.

The catalytic performance of Ag_3_PO_4_ crystals was evaluated in a three-compartment electrochemical cell equipped with gas diffusion electrode (GDE) for the electrooxidation of propylene (Supplementary Fig. 2). We conducted chronoamperometric measurements in 0.1 M PBS (pH = 7.0). After 1-h electrolysis, the catalytic products were determined to be PO, acetone, and acetic acid via ^1^H nuclear magnetic resonance (^1^H NMR) measurements (Supplementary Figs. 3 and 4). At all applied potentials, the faradaic efficiencies (FE) for acetone and acetic acid were lower than 5% (Supplementary Fig. 5). Almost 80% selectivity for PO among liquid products was obtained over Ag_3_PO_4_ cubes, with approximately 75% and 70% selectivity for PO over Ag_3_PO_4_ rhombic dodecahedra and tetrahedra, respectively (Supplementary Fig. 6). As shown in Fig. 2a, Ag_3_PO_4_ cubes exhibited the highest FE for PO (FE_PO_) among Ag_3_PO_4_ crystals at all applied potentials. Especially, at 2.2 V vs RHE, the FE_PO_ over Ag_3_PO_4_ cubes reached 18.7%, whereas the FE_PO_ over Ag_3_PO_4_ rhombic dodecahedra and tetrahedra were 15.9% and 13.1%, respectively. Figure 2b shows the partial current densities (*j*) of PO for Ag_3_PO_4_ crystals. The Ag_3_PO_4_ cubes exhibited higher partial *j* of PO (*j*_PO_) with respect to the other two counterparts at all applied potentials. Notably, the *j*_PO_ over Ag_3_PO_4_ cubes reached the highest value of 0.49 mA cm^−2^ at 2.4 V vs RHE, whereas the *j*_PO_ over Ag_3_PO_4_ rhombic dodecahedra and tetrahedra were 0.31 and 0.19 mA cm^−2^, correspondingly. The Ag_3_PO_4_ cubes exhibited the highest yield rate of 5.3 g_PO_ m^−2^ h^−1^ at 2.4 V vs RHE, which was 1.6 and 2.5 times higher than those over Ag_3_PO_4_ rhombic dodecahedra (3.4 g_PO_ m^−2^ h^−1^) and Ag_3_PO_4_ tetrahedra (2.1 g_PO_ m^−2^ h^−1^) (Fig. 2c). Notably, Ag_3_PO_4_ cubes exhibited a record-high yield rate for PO production among previously reported electrocatalysts for the electrooxidation of propylene (Supplementary Table 2). To explore the intrinsic activity of Ag_3_PO_4_ crystals, we normalized the *j*_PO_ by electrochemical surface area (ECSA). The ECSAs of Ag_3_PO_4_ crystals were determined by measuring double-layer capacitance (*C*_dl_) using cyclic voltammetry (CV) measurements with different scan rates (Supplementary Fig. 7 and Supplementary Table 3). Figure 2d shows the ECSAs-normalized *j*_PO_. At all applied potentials, the ECSA-normalized *j*_PO_ over Ag_3_PO_4_ cubes were always the highest among Ag_3_PO_4_ crystals. The highest ECSA-normalized *j*_PO_ of 0.16 mA cm^−2^ was obtained over Ag_3_PO_4_ cubes at 2.4 V vs RHE, which was 2.3 and 5.3 times as high as those over Ag_3_PO_4_ rhombic dodecahedra (0.07 mA cm^−2^) and Ag_3_PO_4_ tetrahedra (0.03 mA cm^−2^), respectively. Meanwhile, normalized by the ECSA, the commercial Ag_3_PO_4_ exhibited a low ECSA-normalized *j*_PO_ of 0.01 mA cm^−2^ at 2.4 V vs RHE, which was lower than those of the three types of Ag_3_PO_4_ crystals (Supplementary Fig. 8). Accordingly, Ag_3_PO_4_ cubes with exposed (100) facets possessed the highest intrinsic activity for the electrooxidation of propylene.

To investigate the structural stability of Ag_3_PO_4_ cubes during the electrooxidation of propylene, we conducted the in situ X-ray absorption near-edge spectroscopy (XANES) measurements. Based on in situ Ag K-edge XANES profiles, Ag_3_PO_4_ cubes at 2.2 and 2.5 V vs RHE exhibited an energy absorption edge profile in a range from 25,450 to 25,650 eV similar to that of pristine Ag_3_PO_4_ cubes (Supplementary Fig. 9). The durability tests of Ag_3_PO_4_ cubes were proceeded via chronoamperometric measurement for 10 rounds of successive reactions at 2.2 V vs RHE. The FE_PO_ over Ag_3_PO_4_ cubes remained above 17.0% (Supplementary Fig. 10). After durability tests, the SEM images and XRD patterns revealed that the morphology and phase of Ag_3_PO_4_ cubes were perfectly preserved (Supplementary Figs. 11 and 12). The contents of the Ag in the electrolytes and Ag_3_PO_4_ cubes were also determined via inductively coupled plasma atomic emission spectroscopy and X-ray Fluorescence Spectrometer (Supplementary Tables 4 and 5), respectively. These results suggest that Ag_3_PO_4_ cubes are stable during the electrooxidation of propylene. As shown in Supplementary Fig. 13, the contact angle of electrolyte on the GDE of Ag_3_PO_4_ cubes was reduced from 129.9° to 63.4° after ten successive reaction rounds. The transformation of the GDE from hydrophobicity to hydrophilicity would cause the penetration of electrolyte and hinder the diffusion of propylene, resulting in the decreased FE_PO_.

### Reaction paths over Ag_3_PO_4_ crystals

**Fig. 3:**
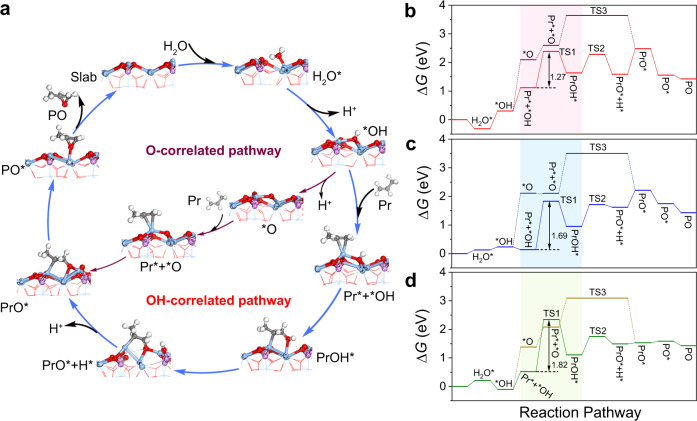
Reaction paths over Ag_3_PO_4_ crystals. **a** Scheme of the two reaction pathways on (100) facets of Ag_3_PO_4_. The gray, white, blue, red, and light pink spheres represent C, H, Ag, O, and P atoms, respectively. **b** Free energy diagram of electrooxidation of propylene with OH-correlated pathway (red) and O-correlated pathway (wine) on (100) facets of Ag_3_PO_4_. **c** Free energy diagram of electrooxidation of propylene with OH-correlated pathway (blue) and O-correlated pathway (navy) on (110) facets of Ag_3_PO_4_. **d** Free energy diagram of electrooxidation of propylene with OH-correlated pathway (green) and O-correlated pathway (chartreuse) on (111) facets of Ag_3_PO_4_. * represents the adsorption site.

To provide a theoretical insight into the reaction mechanism, we carried out DFT calculations by adopting (100), (110), and (111) facets of Ag_3_PO_4_ cubes, Ag_3_PO_4_ rhombic dodecahedra, and Ag_3_PO_4_ tetrahedra as model slabs, respectively (Supplementary Fig. 14). The most stable (100), (110), and (111) facets of Ag_3_PO_4_ crystals possessed different Ag-terminated surfaces via calculating the minimal total energy (Supplementary Table 6). Figure 3a and Supplementary Fig. 15 show the process of electrooxidation of propylene. H_2_O is adsorbed and dissociated into ^*^OH on Ag sites. Afterwards, we considered two reaction pathways classified by adsorbed oxygen species of ^*^OH (OH-correlated pathway) or ^*^O (O-correlated pathway). For OH-correlated pathway, ^*^OH species directly reacts with Pr^*^ to produce a bidentate PrOH^*^ intermediate. Subsequently, the O-H bond in PrOH^*^ is further dissociated to CH_3_CHCH_2_O^*^ (PrO^*^) intermediate, which is an oxametallacycle intermediate during vapor-phase epoxidation of olefin^35,36^. With regard to O-correlated pathway, ^*^OH is further dehydrogenated to ^*^O and couples with Pr^*^ to generate PrO^*^. For the generation of ^*^O, there are two pathways that have been considered^37,38^. As shown in Supplementary Table 7, the change of Gibbs free energy (Δ*G*) for direct dehydrogenation of ^*^OH was lower than that for the disproportionation of ^*^OH over the (100), (110), and (111) facets of Ag_3_PO_4_, respectively. As such, all of the ^*^O are generated from direct dehydrogenation of ^*^OH over the three types of Ag_3_PO_4_ facets. Finally, PrO^*^ is transformed to adsorbed PO (PO^*^) before desorption. The apparent energy barrier is defined as the energy difference between the initial state and the transition state (TS) with the highest energy. As shown in Fig. 3b–d, the apparent energy barriers (initial state→TS1) of OH-correlated pathway are lower than those (initial state→TS3) of O-correlated pathway for each facet of Ag_3_PO_4_ crystals. The electrooxidation of propylene could also undergo the dehydrogenation pathway, but the product would be allyl alcohol, acrolein, and acrylic acid rather than PO according to the previous literatures^21,23^. In addition, the energy barrier for the dehydrogenation of propylene is 1.50 eV on (100) facets of Ag_3_PO_4_, which is higher than that (1.27 eV) for the OH-correlated pathway (Supplementary Fig. 16). To further confirm the OH-correlated pathway with the formation of PrOH^*^ intermediate, we conducted the in situ attenuated total reflection Fourier-transform infrared spectroscopy (ATR-FTIRS) experiment. Supplementary Fig. 17 shows the in situ ATR-FTIRS spectra over the Ag_3_PO_4_ cubes with the applied potentials ranging from 1.0 to 2.6 V vs RHE. The peaks at 1473, 1442, and 1417 cm^−1^ were assigned to the gaseous propylene^39^, which exhibited a decreasing trend as the applied potentials scanning from 1.0 to 2.6 V vs RHE. When the applied potentials were increased, the intensities of the two peaks located at 1541 and 1457 cm^−1^ were also gradually increased. The two characteristic peaks were ascribed to the vibrations of the -C=C- and -CH_3_ in Pr^*^^40,41^, respectively, which was a significant intermediate for the formation of PrOH^*^. Especially, a small peak at 1434 cm^−1^ was observed at potentials higher than 1.6 V vs RHE. The characteristic peak was assigned to the vibration of -CH_2_- in PrOH^*^ intermediate, which was consistent with the calculated peak position of PrOH^*^ species (Supplementary Fig. 17, insert). As such, the electrooxidation of propylene over Ag_3_PO_4_ crystals undergoes the OH-correlated pathway rather than the O-correlated and dehydrogenation pathway.

### Mechanisms of facet effect

**Fig. 4:**
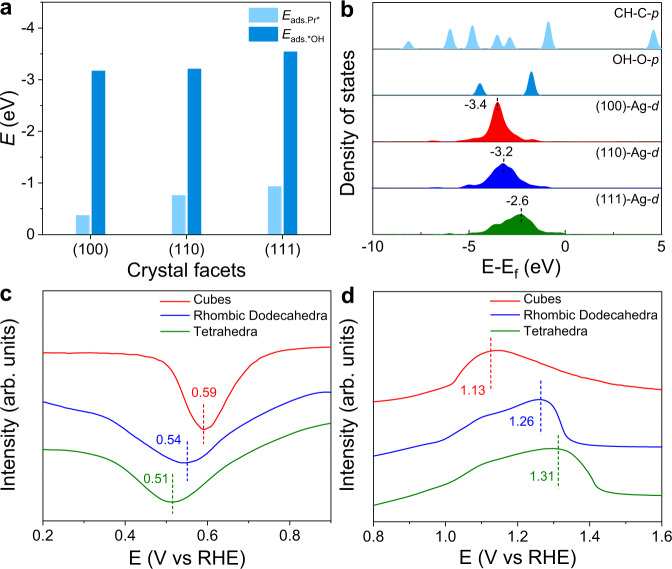
Mechanisms of facet effect. **a** *E*_ads.Pr*_ and *E*_ads.*OH_ over the three types of Ag_3_PO_4_ facets. **b** Projected DOS plots of *d* orbitals of Ag over the three types of Ag_3_PO_4_ facets with *p* orbitals of C in Pr^*^ and O in ^*^OH. The *d*-band center was marked via short dash. **c**, **d** The single reduction of linear sweep voltammetric curves (**c**) and propylene stripping voltammograms (**d**) with the scan rate of 100 mV s^−1^ over Ag_3_PO_4_ crystals.

We measured the apparent activation energies and reaction orders over three types of Ag_3_PO_4_ catalysts. As shown in Supplementary Fig. 18a, the Arrhenius plots of the three types of Ag_3_PO_4_ crystals were obtained. The apparent activation energy of Ag_3_PO_4_ cubes was 8.1 kJ mol^−1^, which was lower than those of Ag_3_PO_4_ rhombic dodecahedra (10.6 kJ mol^−1^) and Ag_3_PO_4_ tetrahedra (15.1 kJ mol^−1^). The reaction order for the electrooxidation of propylene was estimated by plotting the ECSA-normalized *j*_PO_ at 2.2 V vs RHE against the partial pressures of propylene (Supplementary Fig. 18b). For Ag_3_PO_4_ cubes, the reaction order was 0.00, whereas the reaction orders of Ag_3_PO_4_ rhombic dodecahedra and tetrahedra were 0.35 and 0.44, respectively. These results illustrate that the Ag_3_PO_4_ cubes with exposed (100) facets promote the propylene activation, resulting in the high catalytic activity for the electrooxidation of propylene into PO. We also investigated the dependence of RDS on different facets of Ag_3_PO_4_ along the OH-correlated pathway. The formation of PrOH^*^ exhibited the highest reaction energy barrier among all the steps independent of facets of Ag_3_PO_4_ crystals. Thus, the formation of PrOH^*^ serves as the RDS during the electrooxidation of propylene. Specially, the energy barriers of the RDS on (110) and (111) facets of Ag_3_PO_4_ were 1.69 and 1.82 eV, respectively, both higher than that (1.27 eV) on (100) facets of Ag_3_PO_4_. As such, Ag_3_PO_4_ cubes favor the activation of propylene relative to Ag_3_PO_4_ rhombic dodecahedra and Ag_3_PO_4_ tetrahedra (Fig. 3b–d). To rationalize the facet-dependent energy barriers of RDS, we analyzed the adsorption configuration of Pr^*^ and OH^*^ on Ag_3_PO_4_ by comparing the distance of C-O between the C in CH_2_ for Pr^*^ and O for ^*^OH during the RDS. As shown in Supplementary Fig. 19, the (100) facets of Ag_3_PO_4_ exhibited the shortest C-O distance of 1.91 Å in the TS1. The shortened C-O distance benefits the formation of C-O bond, corresponding to the lowered barrier of RDS. Considering that the RDS involved the coupling of Pr^*^ (C) and ^*^OH (O), we further calculated the *E*_ads.Pr*_ and *E*_ads.*OH_^42,43^. As shown in Fig. 4a, the *E*_ads.Pr*_ and *E*_ads.*OH_ on (100) facets of Ag_3_PO_4_ are the lowest among the three types of facets. As such, the energy barrier of the RDS has a positive correlation with the absolute value of *E*_ads.Pr*_ and *E*_ads.*OH_.

To further probe the structure–activity relationship during electrooxidation of propylene over Ag_3_PO_4_, we also analyzed the electronic properties of different Ag sites. Both the bond energies of Ag-C and Ag-O that influenced the *E*_ads.Pr*_ and *E*_ads.*OH_, respectively, were analyzed based on the DOS. Figure 4b shows the projected DOS of these surface Ag atoms on (100), (110), and (111) facets of Ag_3_PO_4_. The *d*-band center of Ag sites on (100) facets shifts away from the Fermi level compared with those on (110) and (111) facets of Ag_3_PO_4_, which exhibits the least overlap with the *p* orbitals of C in Pr^*^ and O in ^*^OH. These results reveal that Ag sites on (100) facets of Ag_3_PO_4_ display the lowest binding energies of both Pr^*^ and ^*^OH^44^. In addition, from the Bader charge analysis, the secondary carbon (CH_2_) and tertiary carbon (CH) are –0.19 |e| and –0.09 |e| charged, respectively, for propylene adsorption on (100) facets of Ag_3_PO_4_ (Supplementary Fig. 20). The difference between these two C atoms is 0.1 |e| whereas the differences on (110) and (111) facets of Ag_3_PO_4_ are 0.07 |e| and 0.01 |e|, respectively. In this case, the polarization of propylene on (100) facets of Ag_3_PO_4_ was the largest among all the facets. The polarization of propylene and negative secondary carbon atoms broke the symmetric π bonding and facilitated the formation of C-O bond.

To gain insight into the facet-dependent binding energies of ^*^OH and Pr^*^, we conducted the OH^−^ and propylene stripping experiments^21,45–47^. As shown in Fig. 4c, the reduction peaks of surface hydroxyl intermediates shifted from 0.59 V vs RHE (Ag_3_PO_4_ cubes) to 0.54 V (Ag_3_PO_4_ rhombic dodecahedra) and 0.51 V vs RHE (Ag_3_PO_4_ tetrahedra), respectively. These results suggest that the binding energy of ^*^OH on Ag_3_PO_4_ cubes is the lowest among all the samples. Figure 4d shows the propylene stripping profiles over Ag_3_PO_4_ crystals. A broad peak at around 1.13 V vs RHE was observed over Ag_3_PO_4_ cubes. Meanwhile, the stripping peaks of propylene over Ag_3_PO_4_ rhombic dodecahedra and tetrahedra were located at around 1.26 and 1.31 V vs RHE. The positive shift of the peak potential manifested that the adsorbed strength of Pr^*^ over Ag_3_PO_4_ cubes was lower than those over Ag_3_PO_4_ rhombic dodecahedra and Ag_3_PO_4_ tetrahedra. Both DFT calculations and experiments proved that *E*_ads.Pr*_ and *E*_ads.*OH_ over Ag_3_PO_4_ cubes were the lowest, which was consistent with the highest activity for the electrooxidation of propylene.

## Discussion

We achieved highly efficient electrocatalysts of Ag_3_PO_4_ cubes which performed a high yield rate (5.3 g_PO_ m^−2^ h^−1^) of PO production for the electrooxidation of propylene. Moreover, we demonstrated the facet effect from two aspects. On the one hand, the DOS of (100) facets on Ag_3_PO_4_ exhibited the least overlap with the *p* orbitals of C in Pr^*^ and O in ^*^OH, thus displaying the lowest *E*_ads.Pr*_ and *E*_ads.*OH_. The weakened adsorption of Pr^*^ and ^*^OH lowered the energy barrier of PrOH^*^ formation that was determined as the RDS, accounting for the enhanced activity. On the other hand, the polarization of propylene on (100) facets of Ag_3_PO_4_ was the largest among all the facets from the Bader charge analysis, which was conducive to breaking the symmetric π bonding and facilitating the formation of C-O bond. Our work not only offers an effective catalyst for the electrooxidation of propylene but also advances the understanding of the facet effect on catalytic performance.

## Methods

### Chemicals and materials

Ammonium nitrate (NH_4_NO_3_, 99%), silver nitrate (AgNO_3_, 99.8%), sodium hydroxide (NaOH, 99%), potassium hydrogen phosphate (K_2_HPO_4_, 99.5%), ethanol (EtOH, 99.8%) were all purchased from Sinopharm Chemical Reagent Co. Ltd. (Shanghai, China). 1-Propanesulfonic acid 3-(trimethylsilyl) sodium salt (DSS), Dimethyl Sulfoxide-d6 (99.9 atom % D, contains 0.03% v/v TMS), Nafion solution (5 wt%) and Nafion 115 film were purchased from Sigma-Aldrich. PO (99.5%), acetone (99.5%), acetic acid (99.5%), and commercial Ag_3_PO_4_ were purchased from Aladdin Co. Ltd. (Shanghai, China). The deionized (DI) water with a resistivity of 18.2 MΩ cm was provided by a Millipore Milli-Q grade. All of the chemicals were used without any further purification.

### Synthesis of Ag_3_PO_4_ cubes

In a typical procedure, 89.2 mL of DI water was added to the beaker, then 1 mL of NH_4_NO_3_ solution (0.4 M), 1.8 mL of NaOH solution (0.2 M), and 4 mL of AgNO_3_ solution (0.05 M) were added to the beaker sequentially. The solution was stirred vigorously for 10 min to prepare the [Ag(NH_3_)_2_]^+^ complex. Finally, 4 mL of K_2_HPO_4_ solution (0.1 M) was added to the complex and stirred for 5 min. After the solution color turned from colorless to light yellow, Ag_3_PO_4_ cubes were obtained. The as-obtained precipitate was separated by centrifugation and washed subsequently with DI water three times.

### Synthesis of Ag_3_PO_4_ rhombic dodecahedra

Except for the feeding ratio of the reactants, the synthetic method of rhombic dodecahedra was similar to that of cubes. Specifically, 89.2 mL of DI water was replaced by 84.2 mL of DI water. 1 mL of NH_4_NO_3_ solution (0.4 M) was substituted by 6 mL of NH_4_NO_3_ solution (0.4 M). The other steps were the same as those of cubes.

### Synthesis of Ag_3_PO_4_ tetrahedra

Three mmol of AgNO_3_ was dissolved in 30 mL of ethanol under rapid stirring for the formation of AgNO_3_-ethanol solution. Simultaneously, 5 mL of H_3_PO_4_ was mixed with 30 mL of ethanol for the formation of H_3_PO_4_-ethanol solution. Then, the AgNO_3_-ethanol solution was added dropwise to the H_3_PO_4_-ethanol solution, until the mixture turned slightly cloudy. Finally, the mixture was added back into the AgNO_3_-ethanol solution. After 1-h stirring, the solution turned bright green. The as-obtained precipitate was separated by centrifugation and washed subsequently with ethanol three times.

### Preparation of working electrodes

For all Ag_3_PO_4_ crystals, the as-prepared sample and Nafion solution were ultrasonically suspended in ethanol and uniformly spread on carbon papers with a loading amount of 2.5 mg cm^−2^.

### Electrochemical measurements

For the electrooxidation of propylene, the electrochemical measurements were carried out in a three-compartment electrochemical cell equipped with a GDE. Graphite rod and Ag/AgCl electrodes were used as the counter electrode and reference electrode, respectively. The potentials were controlled by an Autolab potentiostat/galvanostat (CHI 660E). All potentials were measured against the Ag/AgCl reference electrode and converted to the RHE reference scale on account of the equation:

1 $$E({{{{{\rm{vs}}}}}}\,{{{{{\rm{RHE}}}}}})=E({{{{{\rm{vs}}}}}}\,{{{{{\rm{Ag}}}}}}/{{{{{\rm{AgCl}}}}}})+0.21{{{{{\rm{V}}}}}}+0.0591\times {{{{{\rm{pH}}}}}}$$

After propylene was purged into the gas cavity for 300 s to remove the residual air, chronoamperometric electrolysis was performed at each potential for 1 h. The liquid products in the electrolyte were quantified via ^1^H NMR analysis. The FE_PO_ was calculated at a given potential as follows:

2 $${{{{{\rm{FE}}}}}}=c\times V\times N\times F/Q$$

where *c* represents the concentration of product for PO, *V* represents the volume of the electrolyte, *N* represents the number of electrons transferred for product formation, which is 2 for PO, *F* is the Faraday constant, and *Q* represents the quantity of electric charge integrated by *i*–*t* curve.

The CV measurements were conducted in 0.1 M PBS solution using a three-electrode cell equipped with an Ag/AgCl reference electrode and a graphite counter electrode. 10 mg of the catalyst was dispersed in a mixture of 950-μL ethanol and 50-μL Nafion solution under ultrasonic stirring to form a homogenous ink. Ten microliter of the ink was then dropped onto a glassy carbon disk electrode by a micropipette to form a catalyst layer. CVs of Ag_3_PO_4_ crystals were conducted with various scan rates (10, 20, 30, 40, 50, 60, 70, 80, 90, and 100 mV s^−1^) under argon atmosphere to obtain the double-layer capacitance (*C*_dl_). The *C*_dl_ was estimated by plotting the Δ*j* (*j*_a_–*j*_c_) in the middle of the scan range against the scan rates, where *j*_a_ and *j*_c_ were the anodic and cathodic current density, respectively. The linear slope was equivalent to twice of the *C*_dl_. The ECSAs was calculated by the following equation:

3 $${{{{{\rm{ECSAs}}}}}}={R}_{{{{{{\rm{f}}}}}}}{{{{{\rm{S}}}}}}$$

where *R*_f_ represented the roughness factor of Ag_3_PO_4_ surface and S represented the surface area of carbon paper electrode (1 cm^2^ in this case). Based on the *C*_dl_ of a smooth oxide surface (60 μF cm^−2^ for Ag_3_PO_4_ surface^48^), *R*_f_ was calculated according to the relation *R*_f_ = *C*_dl_/60.

### Instrumentations

XRD patterns were recorded by using a Philips X’Pert Pro Super diffractometer with Cu-*K*α radiation (λ = 1.54178 Å). XPS measurements were performed on a VG ESCALAB MK II X-ray photoelectron spectrometer with an exciting source of Al *Kα* = 1486.6 eV. The liquid products were examined on a Varian 400 MHz NMR spectrometer (Bruker AVANCE AV III 400). SEM images were taken using a Hitachi SU8220 scanning electron microscope. TEM images were taken using a Hitachi H-7650 transmission electron microscope at an acceleration voltage of 100 kV. SAED were carried out on a JEOL ARM-200F field-emission transmission electron microscope operating at an accelerating voltage of 200 kV using Cu-based TEM grids. Inductively coupled plasma atomic emission spectroscopy (Atomscan Advantage, Thermo Jarrell Ash, USA) was conducted to determine the concentration of Ag species. X-Ray Fluorescence Spectrometer (XRF-1800, SHIMADZU) was used to qualify the molar ratio of Ag to P for the GDE of Ag_3_PO_4_ cubes. The in situ ATR-FTIRS measurements were carried out on a Nicolet iS50 with a wavenumber resolution of 4 cm^−1^ at room temperature.

### In situ XANES measurements

In situ XANES experiments were carried out at BL14W1 beamline of Shanghai Synchrotron Radiation Facility. The XANES spectra of Ag K*-*edge (*E*_0_ = 25514 eV) were operated at 3.5 GeV under “top-up” mode with a constant current of 260 mA. The in situ XANES data were recorded under fluorescence mode with a H-cell. The electrolyte was propylene-saturated 0.1 M PBS (pH = 7), while Ag/AgCl and a graphite rod acted as a reference and counter electrodes, respectively. The working electrode was prepared with a loading amount of 2.5 mg cm^−2^ for Ag_3_PO_4_ cubes. The energy was calibrated accordingly to the absorption edge of pure Ag foil. For the X-ray absorption near-edge structure part, the experimental absorption coefficients as a function of energies μ(E) were processed by background subtraction and normalization procedures, which were reported as “normalized absorption”.

### Computational methods

The Vienna ab initio Simulation Package was implemented in the DFT calculation in this work^49^. The projector augmented wave method and Perdew–Burke–Ernzerhof generalized gradient approximation method were used to describe the inner cores and the contributions of exchange-correlation functional, respectively^50,51^. The total energy calculations were performed using a 3 × 3 × 1 grid and a plane wave cut-off energy of 400 eV. The (100) facet of Ag_3_PO_4_ contained three unit layers (12 atomic layers) with a supercell formula of Ag_36_P_12_O_48_. The (110) facet of Ag_3_PO_4_ contained two layers with a supercell formula of Ag_24_P_8_O_32_. The (111) facet of Ag_3_PO_4_ contained three Ag_3_PO_4_ layers with a supercell formula of Ag_36_P_12_O_48_. A vacuum space of 12 Å was added in Z-direction to avoid the interactions between each slab. Atoms in the bottom Ag_3_PO_4_ layer were fixed and all other atoms including adsorbates were allowed to relax until the force on each ion was smaller than 0.02 eV/Å.

The Gibbs free energy changes (Δ*G*) for the elementary steps in the proposed mechanism are calculated as follows:

4 $$\varDelta G=\varDelta {E}_{{{{{{\rm{DFT}}}}}}}+\varDelta {{{{{\rm{ZPE}}}}}}-T\varDelta S$$

Here, Δ*E*_DFT_ is the change of energies of the optimized structures from DFT calculations. ΔZPE is the zero-point energy difference by analyzing the frequencies. Δ*S* is the entropy of each intermediate. It is assumed that *S* = 0 for all the adsorbed species. TSs of key elementary steps were searched using the climbing image nudged elastic band method^52^. All TSs were confirmed as true saddle points with a single imaginary frequency mode along the reaction coordinate. According to the model of computational hydrogen electrode, at standard conditions, the free energy change of the surface deprotonation process is equivalent to the hydrogen production, namely ^*^H + e^−^ = 1/2 H_2_^53^. The adsorption energy (*E*_ads_) is defined as follows:

5 $${E}_{{{{{{\rm{ads}}}}}}}={E}_{{{{{{\rm{adsorb}}}}}}/{{{{{\rm{surf}}}}}}}-{E}_{{{{{{\rm{surf}}}}}}}-{E}_{{{{{{\rm{adsorb}}}}}}}$$

where *E*_adsorb/surf_, *E*_surf_, and *E*_adsorb_ represent the total energies of the slab with adsorbate(s), the clean slab, and the isolated adsorbate, respectively.

## Supplementary information

Supplementary Information

## Data Availability

All the data supporting this study are available in the paper and Supplementary Information. Source Data are provided with this paper.

## Source data

Source Data

## Code availability

The codes and simulation files that support the plots and data analysis within this paper are available from the corresponding author upon reasonable request.
