# Supplementary material for: Facet-dependent electrooxidation of propylene into propylene oxide over Ag3PO4 crystals
Source: Nat Commun. 2022 Feb 17;13:932. doi: 10.1038/s41467-022-28516-0 (PMC8854733; doi:10.1038/s41467-022-28516-0)
Supplement: Supplementary file 1 — Supplementary Information [file 41467_2022_28516_MOESM1_ESM.pdf]

Supplementary Information for

Facet-Dependent Electrooxidation of Propylene into Propylene Oxide over Ag_3PO_4 Crystals

Jingwen Ke,[1,2] Jiankang Zhao,[1,2] Mingfang Chi,[1,2] Menglin Wang,[1] Xiangdong Kong,[1] Qixuan Chang,[1] Weiran Zhou,[1] Chengxuan Long,[1] Jie Zeng,[1] Zhigang Geng[1]*

[1]Hefei National Laboratory for Physical Sciences at the Microscale, Key Laboratory of Strongly-Coupled Quantum Matter Physics of Chinese Academy of Sciences, Key Laboratory of Surface and Interface Chemistry and Energy Catalysis of Anhui Higher Education Institutes, Department of Chemical Physics, University of Science and Technology of China, Hefei, Anhui 230026, P. R. China

[2]These authors contributed equally to this work.

*Corresponding author. Email: gengzg@ustc.edu.cn (Z.G.).

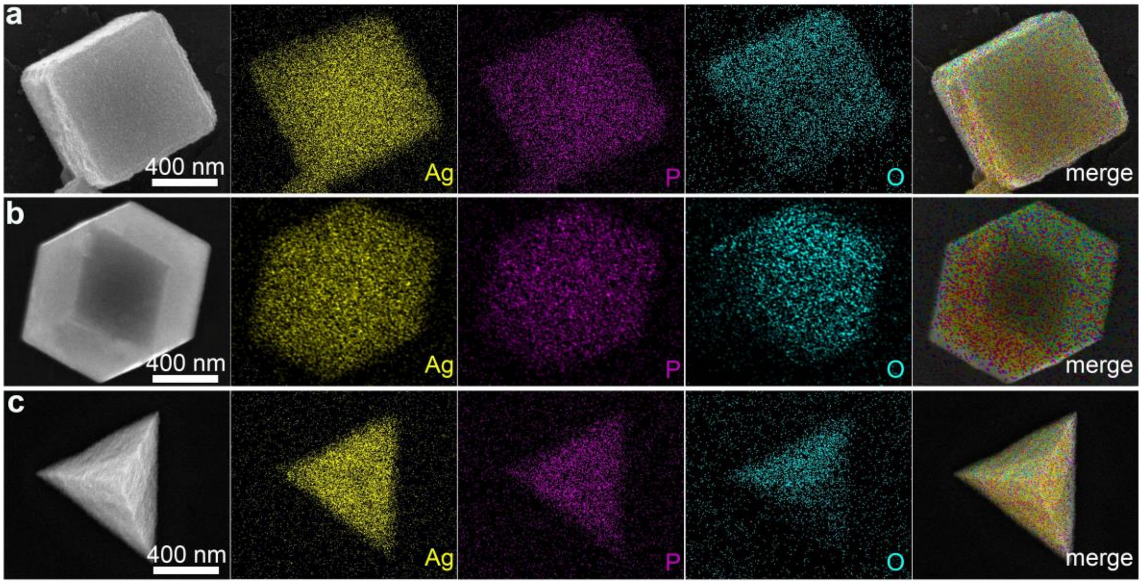

Supplementary **Figure 1.** Scanning electron microscopy-energy dispersive X-ray elemental mapping of the Ag_3PO_4 cube (**a**), rhombic dodecahedron (**b**), and tetrahedron (**c**).

Supplementary **Table 1.** The intensity ratio of the (200), (110) and (222) diffractions relative to (222) diffractions for the Ag_3PO_4 crystals.

Morphology	Intensity ratio		
	(200)/(222)	(110)/(222)	(222)/(222)
Cubes	1.85	0.99	1.00
Rhombic dodecahedra	0.78	2.55	1.00
Tetrahedra	0.36	0.15	1.00
Standard	1.29	1.00	1.00

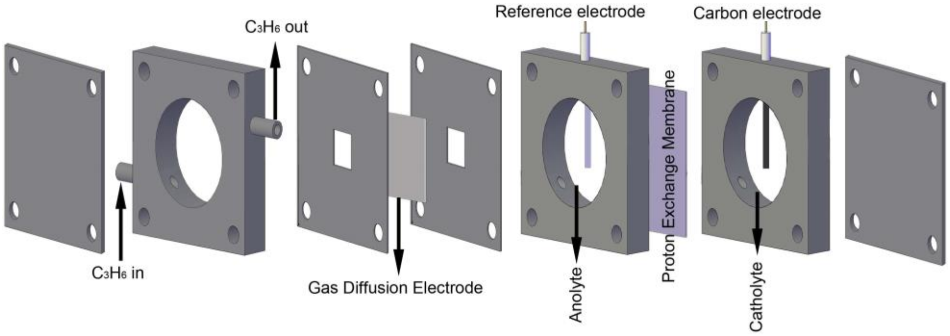

Supplementary **Figure 2.** Schematic illustration of the three-compartment electrochemical cell equipped with gas diffusion electrode.

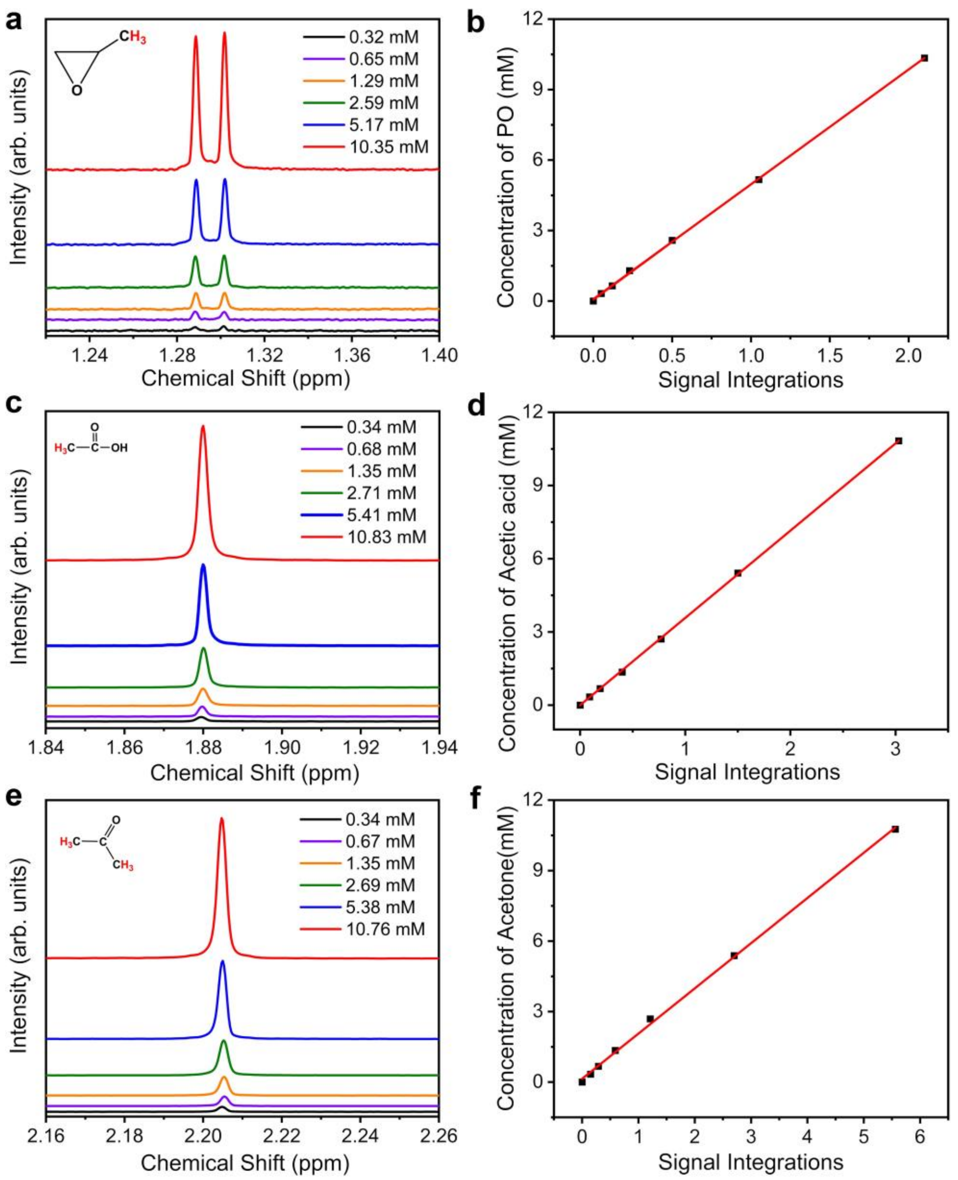

Supplementary **Figure 3. ^{1}H NMR measurements for PO, acetic acid, and acetone solutions**. ^{1}H NMR spectra of PO (**a**), acetic acid (**c**), and acetone (**e**) solutions with a series of standard concentrations. The corresponding concentration-integral area curves of PO (**b**), acetic acid **(d)**, and acetone **(f)** solutions.

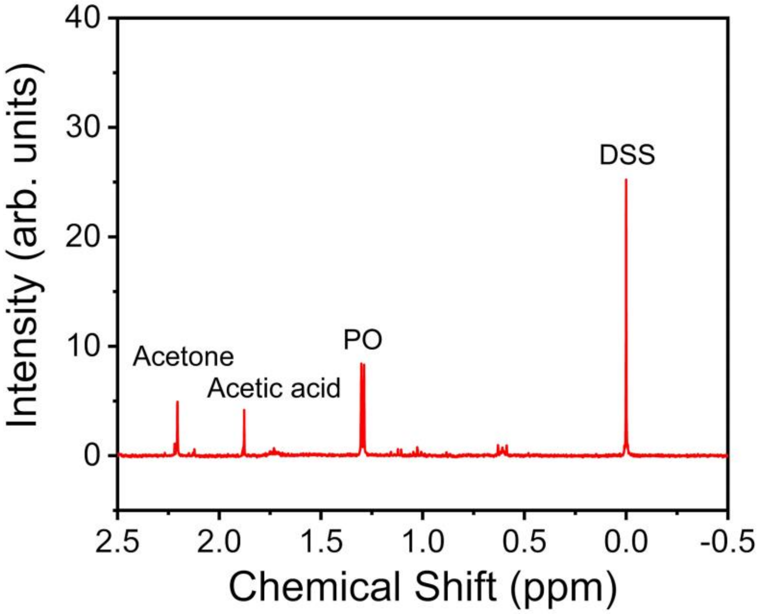

Supplementary **Figure 4.** ^{1}H NMR spectrum of the liquid products over Ag_3PO_4 cubes acquired *via* 1-h chronoamperometric test at 2.2 V vs RHE.

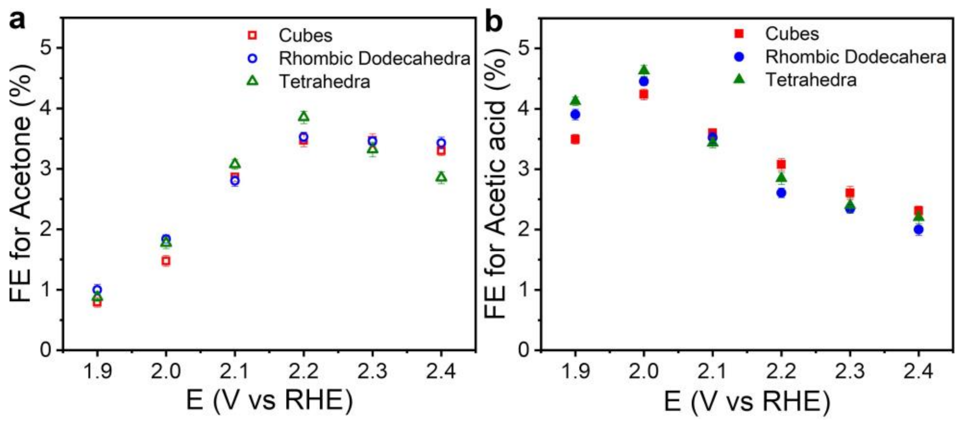

Supplementary **Figure 5.** FE for acetone (**a)** and acetic acid (**b)** over Ag_3PO_4 crystals at all applied potentials. The error bars represent the standard deviations for the three independent measurements.

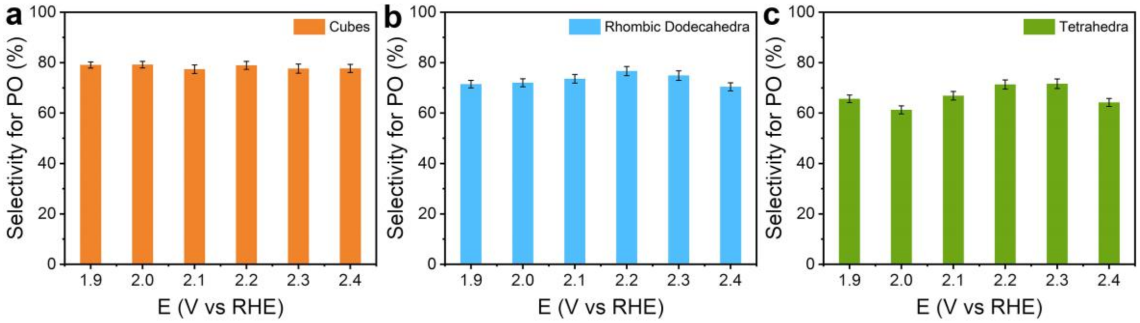

Supplementary **Figure 6.** Selectivity for PO production among liquid products over Ag_3PO_4 cubes **(a)**, rhombic dodecahedra **(b)**, and tetrahedra **(c)**. The selectivity for PO was calculated *via* the molar amount of PO divided by the molar amount of all liquid products. The error bars represent the standard deviations for the three independent measurements.

Supplementary **Table 2.** Comparison of the catalytic performance for Ag_3PO_4 crystals with previously reported electrocatalysts.

Catalysts	Electrolyte	Potential	Yield rate		Ref
			Area activity (g_{po} m^{-2} h^{-1})	Mass activity (g_{po} g_{cat}^{-1} h^{-1})	
Ag sheets	~0.01 M NaOH	/	< 0.01	/	[1]
PtO_2/C	1 M H_3PO_4	2.0 V cell voltage	/	0.05	[2]
Tetrahedra	1M PBS	2.3 V vs RHE	2.56	0.10	this work
Rhombic dodecahedra	1M PBS	2.4 V vs RHE	3.39	0.14	this work
Cubes	1M PBS	2.4 V vs RHE	5.33	0.21	this work

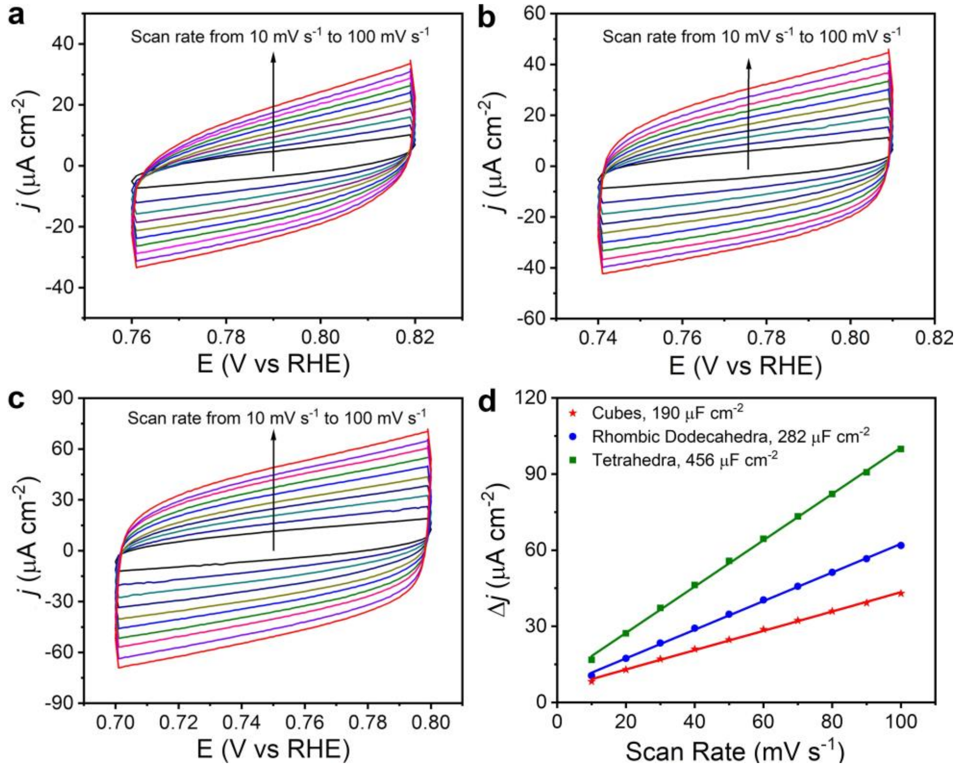

Supplementary **Figure 7.** CV curves were performed at various scan rates over Ag_3PO_4 cubes (**a**), rhombic dodecahedra (**b**), and tetrahedra (**c**). (**d**) Charging current density differences plotted against scan rates over the Ag_3PO_4 crystals.

Supplementary **Table 3.** The ECASs for the Ag_3PO_4 catalysts.

Morphology	C_{dl}	$R_f = C_{dl}/60$	ECSA = $R_f S$
Cubes	190 μF cm^{-2}	3.17	3.17 cm^2
Rhombic Dodecahedra	282 μF cm^{-2}	4.70	4.70 cm^2
Tetrahedra	456 μF cm^{-2}	7.60	7.60 cm^2

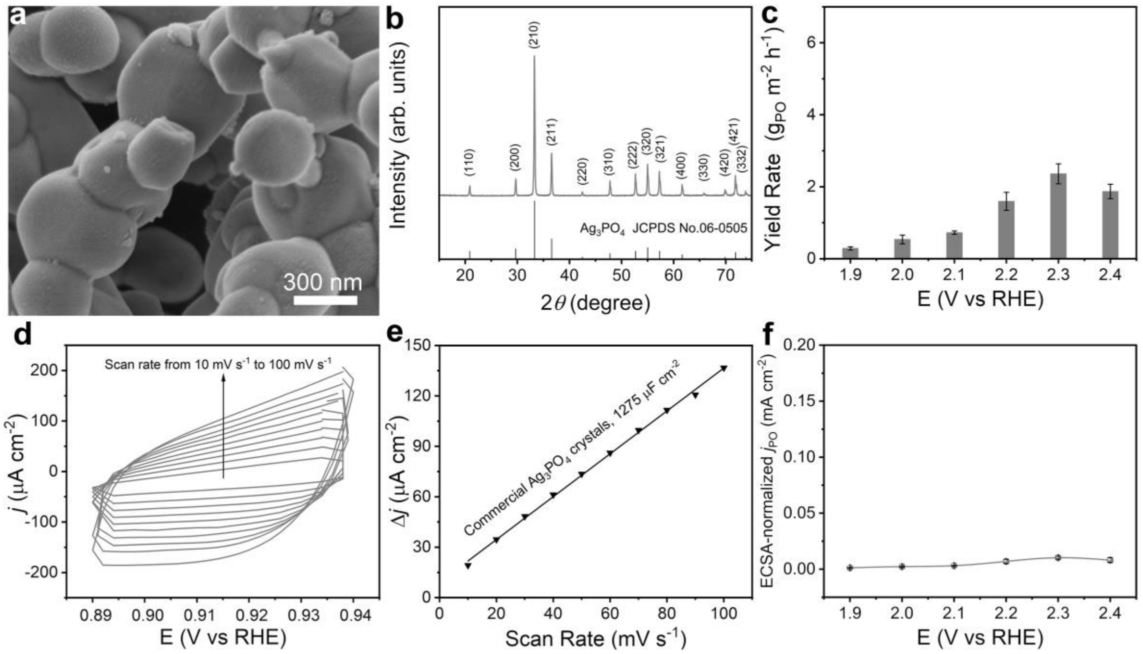

Supplementary **Figure 8**. **Structural characterizations and Catalytic performance of commercial Ag_3PO_4 crystals.** SEM image (**a**) and XRD pattern (**b**) of commercial Ag_3PO_4 crystals. Yield rates of PO normalized by the geometric area of electrode (**c**), CV curves performed at various scan rates (**d**), Charging current density differences plotted against scan rates (**e**), and ECSA-normalized j_{PO} (f) over commercial Ag_3PO_4 crystals. The error bars represent the standard deviations for the three independent measurements.

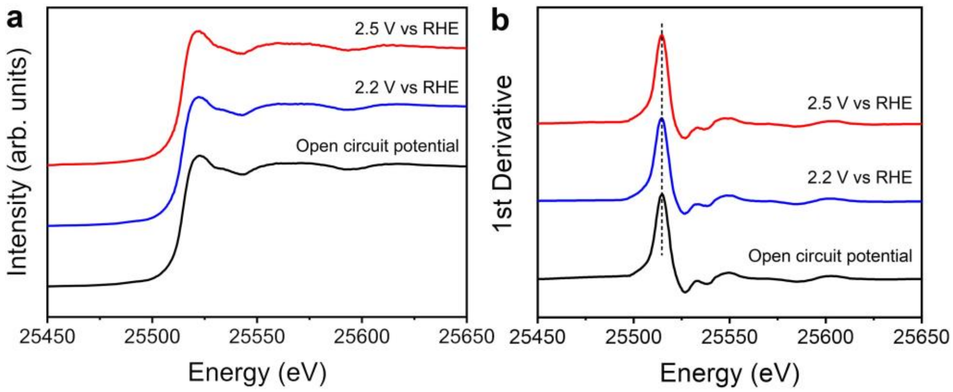

Supplementary **Figure 9.** (a) *In-situ* XANES of Ag_3PO_4 cubes at open circuit potential and Ag_3PO_4 cubes at 2.2 and 2.5 V vs RHE. (b) The differential of the three plots from the *in-situ* XANES of Ag_3PO_4 cubes.

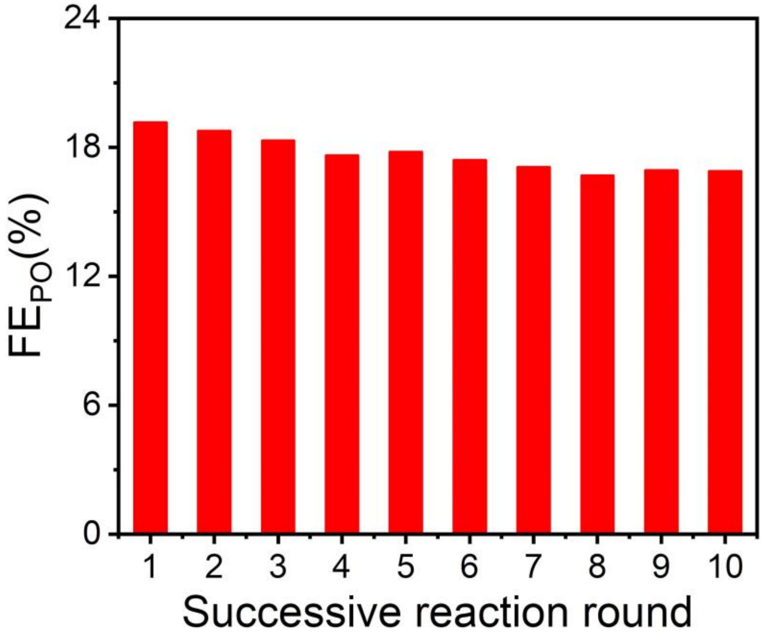

Supplementary **Figure 10.** The FE_{PO} over Ag_3PO_4 cubes for 10 rounds of successive reactions at 2.2 V vs RHE.

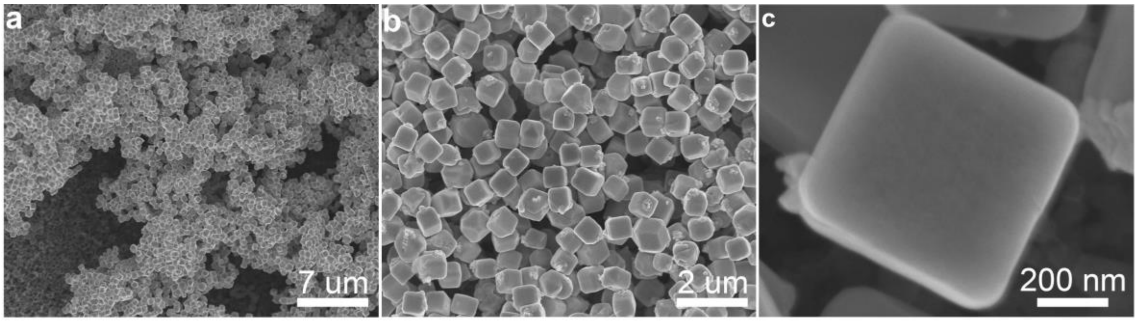

Supplementary **Figure 11.** The morphology of Ag_3PO_4 cubes after 10 rounds of successive reactions at 2.2 V vs RHE.

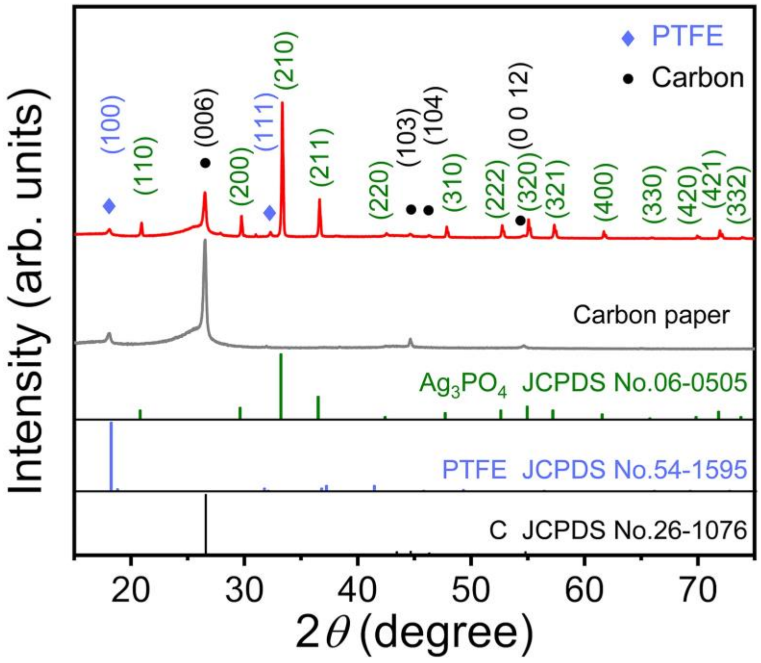

Supplementary **Figure 12.** The XRD patterns for the GDE of Ag_3PO_4 cubes after 10 rounds of successive reactions at 2.2 V vs RHE. The small peaks located at 18.1 ° and 32.0 ° were attributed to (100) and (111) facets of the polytetrafluoroethylene (JCPDS No. 54-1595) in commercial carbon paper. The peaks at 26.6 °, 44.7 °, 46.3 °, and 54.8 ° were assigned to (006), (103), (104), and (0 0 12) facets of graphite carbon (JCPDS No. 26-1076).

Supplementary **Table 4.** ICP-AES measurements of the electrolytes (0.1 M PBS) before and after the successive reactions at 2.2 V vs RHE.

Samples	Ag (ppm)
Fresh PBS	0.018
PBS after electrolysis	0.019

Supplementary **Table 5.** The molar ratios of Ag to P for Ag_3PO_4 Cubes before and after ten successive reaction rounds. The commercial Ag_3PO_4 was conducted as the standard sample.

Samples	molar ratio of Ag/F
Standard Ag_3PO_4	3.00 ± 0.01
Ag_3PO_4 Cubes	3.01 ± 0.03
Ag_3PO_4 Cubes after electrolysis	2.98 ± 0.04

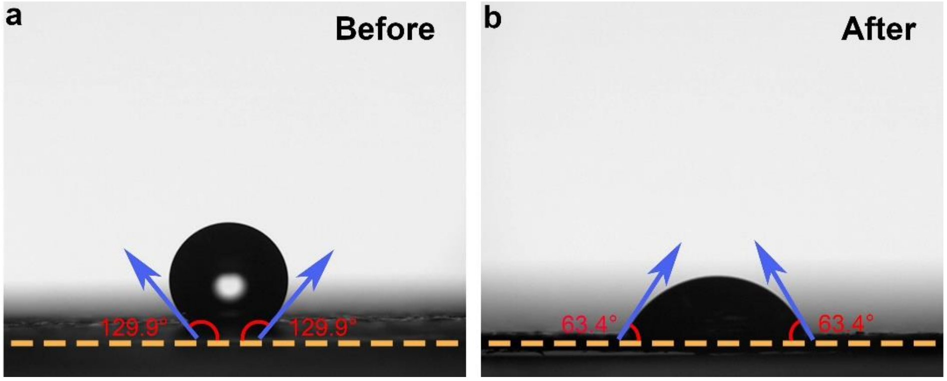

Supplementary **Figure 13.** Contact angles of electrolyte on the GDE of Ag_3PO_4 cubes before (**a**) and after (**b**) the ten successive reaction rounds.

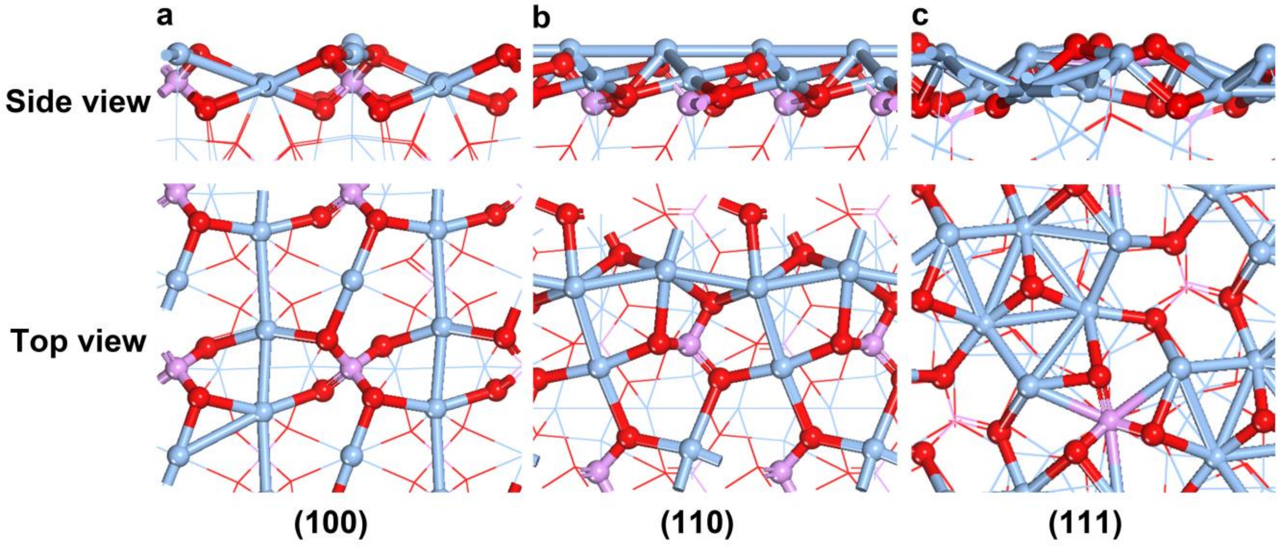

Supplementary **Figure 14.** The most stable structures of (100) (**a**), (110) (**b**), and (111) (**c**) facets of Ag_3PO_4. The blue, red, and light pink spheres represent Ag, O, and P atoms, respectively.

Supplementary **Table 6.** The total energies of the Ag_3PO_4 slabs (E_{slab}) with different terminated atoms on (100), (110), and (111) facets, respectively.

(100) facets		(110) facets		(111) facets	
Top layers	E_{slab} (eV)	Top layers	E_{slab} (eV)	Top layers	E_{slab} (eV)
Ag, P-1st; O-2nd	-461.97	P, O-1st; Ag, O-2nd	-161.30	P-1st; Ag, O-2nd	-472.10
O-1st; Ag, P-2nd	-499.27	Ag-1st; P, O-2nd	-157.91	O-1st; P, O-2nd	-493.51
Ag-1st; O-2nd	-499.64	Ag, O-1st; P, O-2nd	-165.78	Ag-1st; O-2nd	-493.50
O-1st; Ag-2nd	-471.54	Ag-1st; Ag, O-2nd	-165.84	/	/

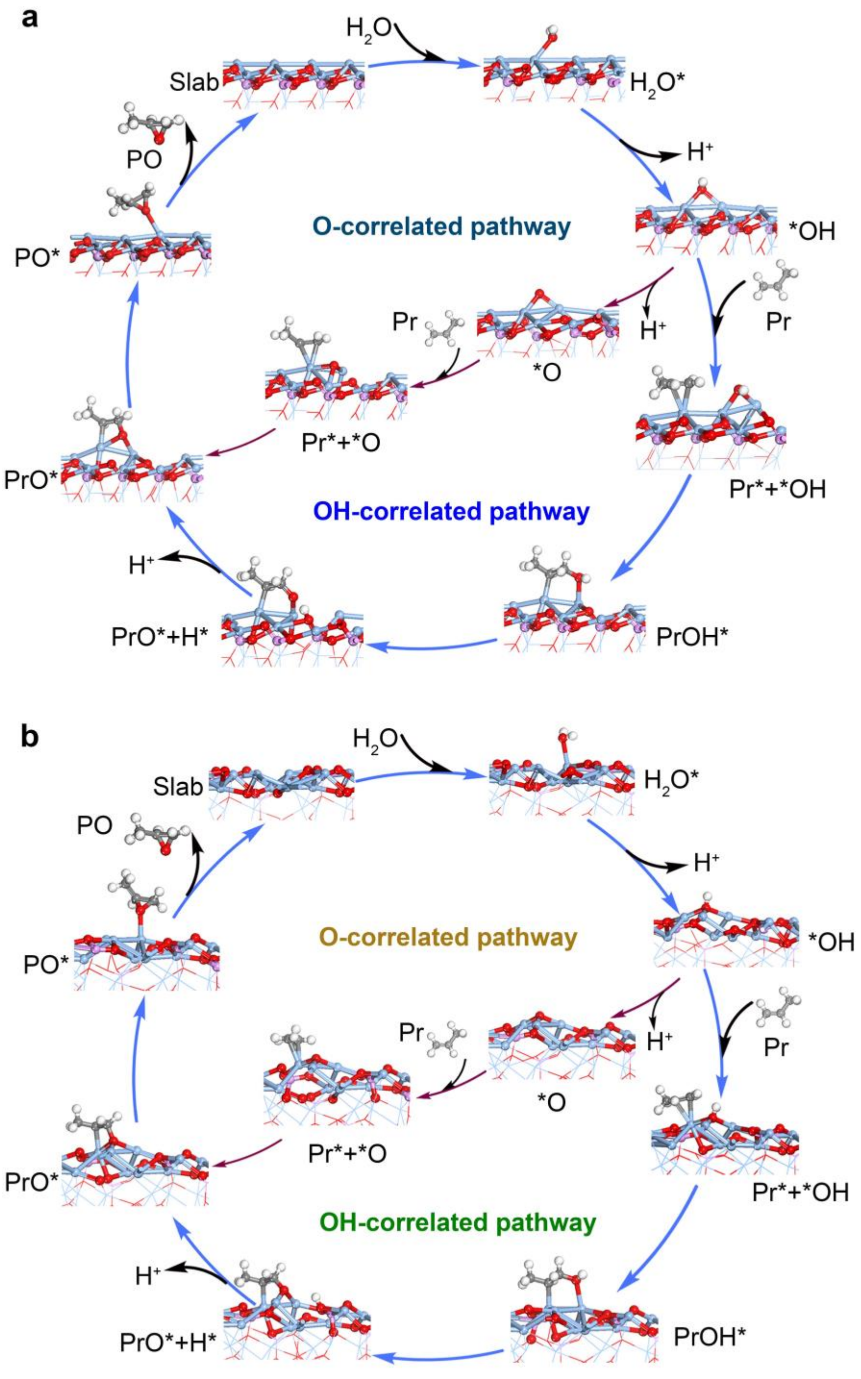

Supplementary **Figure 15.** Scheme of the two reaction pathways for the electrooxidation of propylene on (110) (**a**) and (111) (**b**) facets of Ag_3PO_4. The gray, white, blue, red, and light pink spheres represent C, H, Ag, O, and P atoms, respectively.

Supplementary **Table 7.** The ΔG of the two pathways for the *OH to *O on (100), (110), and (111) facets of Ag_3PO_4, respectively.

Elementary steps	ΔG (eV)		
	(100) facets	(110) facets	(111) facets
$^*OH \rightarrow {}^*O + H^*$	0.37	0.74	1.17
$^*OH + {}^*OH \rightarrow {}^*O + H_2O^*$	0.87	1.35	1.53

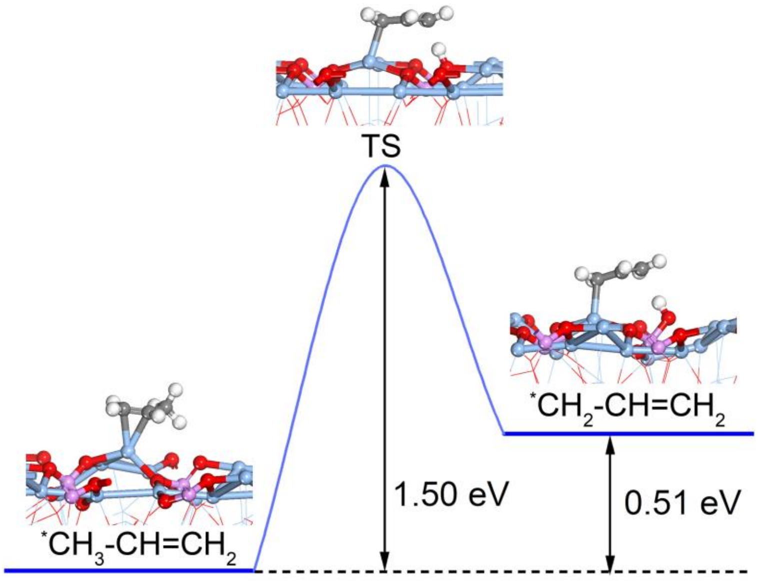

Supplementary **Figure 16.** Free energy diagram of the pathway for the dehydrogenation of propylene on (100) facets of Ag_3PO_4.

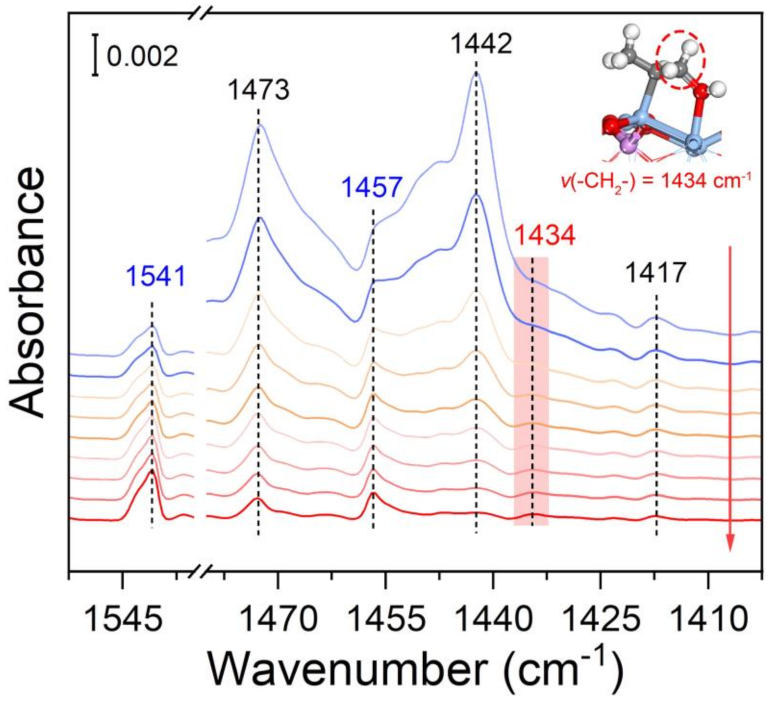

Supplementary **Figure 17**. *In-situ* ATR-FTIR spectra recorded at applied potentials scanning from 1.0 V to 2.6 V vs RHE (following the red arrow) over Ag_3PO_4 cubes towards propylene electrooxidation. The calculated vibrational frequency of the $-CH_2-$ in the $PrOH^*$ was shown in the insert.

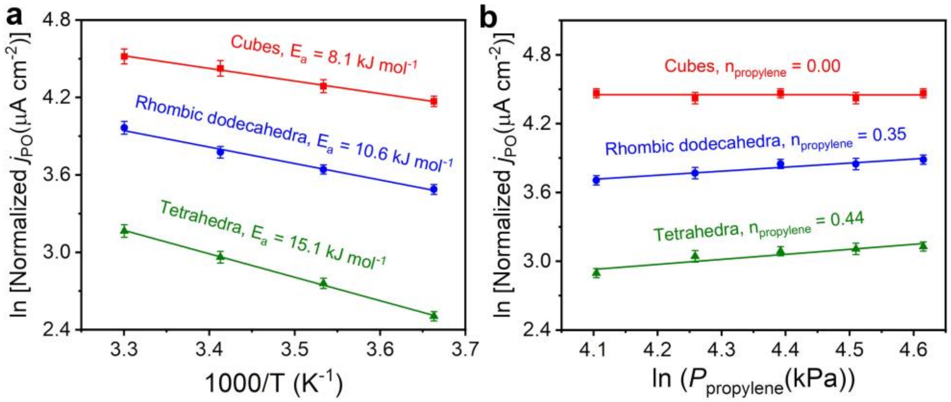

Supplementary **Figure 18.** (**a**) Arrhenius plot of the ECSA-normalized j_{PO} over the three types of Ag_3PO_4 crystals at the temperature from 273 to 303 K. The fitting curves show the linear relation over the three types of Ag_3PO_4 crystals (Ag_3PO_4 cubes: y = -0.98x + 7.75, R^2 = 0.996; Ag_3PO_4 rhombic dodecahedra: y = -1.28x + 8.15, R^2 = 0.988; Ag_3PO_4 tetrahedra: y = -1.82x + 9.17, R^2 = 0.998). (**b**) Effect of propylene partial pressure on ECSA-normalized j_{PO} over the three types of Ag_3PO_4 crystals. The error bars represent the standard deviations for the three independent measurements.

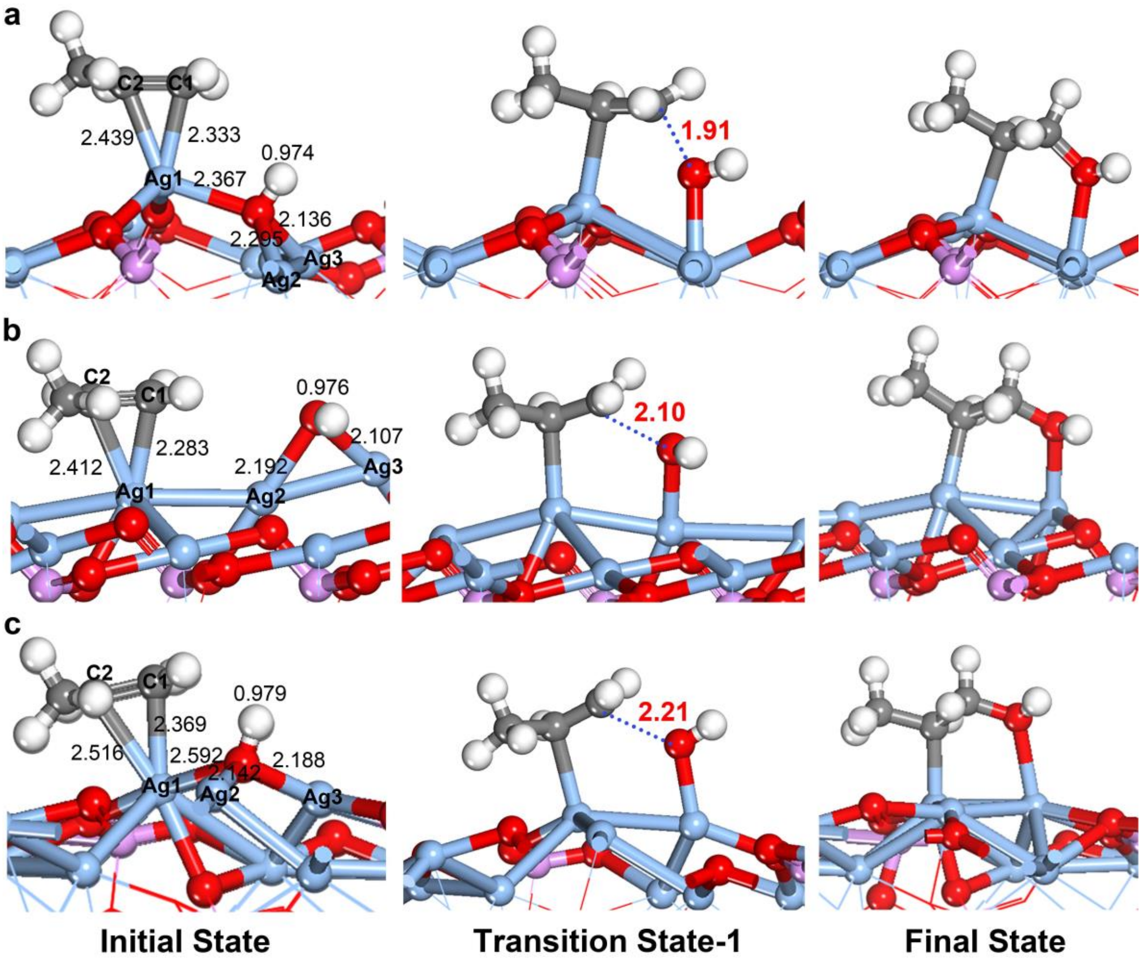

Supplementary **Figure 19.** The distance of C-O between C in CH_2 for Pr* and O for *OH in Transition state-1 (TS1) on (100) (**a**), (110) (**b**), and (111) (**c**) facets of Ag_3PO_4. The gray, white, blue, red, and light pink spheres represent C, H, Ag, O, and P atoms, respectively. The unit of distance marked in the graphs is Å.

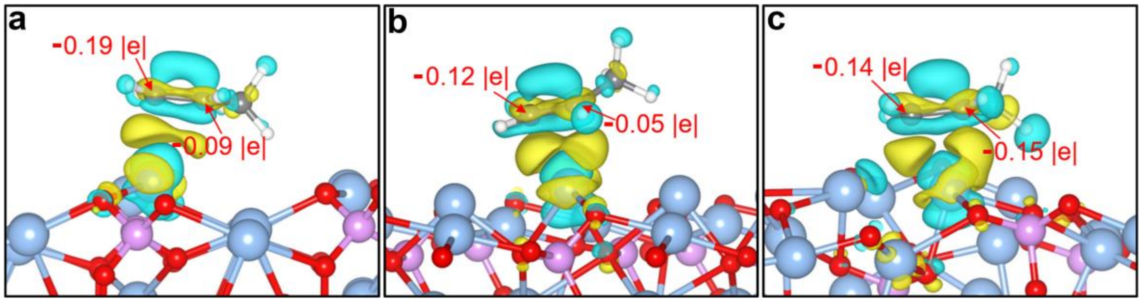

Supplementary **Figure 20.** The charge density difference of propylene adsorption on (100) (**a**), (110) (**b**) and (111) (**c**) facets of Ag_3PO_4 with the isosurface value of 0.002 e/Bohr^3. The gray, white, blue, red, and light pink spheres represent C, H, Ag, O, and P atoms, respectively. The yellow and light blue represent the charge accumulation and depletion, respectively.

Supplementary References

1. Holbrook, L. L. & Wise, H. Electrooxidation of olefins at a silver electrode. *J. Catal.* **38**, 294-298 (1975).

2. Yamanaka, I., Sato, K. & Otsuka, K. Electrolytic synthesis of propene oxide from propene and water in the Gas Phase. *Electrochem. Solid-State Lett.* **2**, 131-132 (1999).
